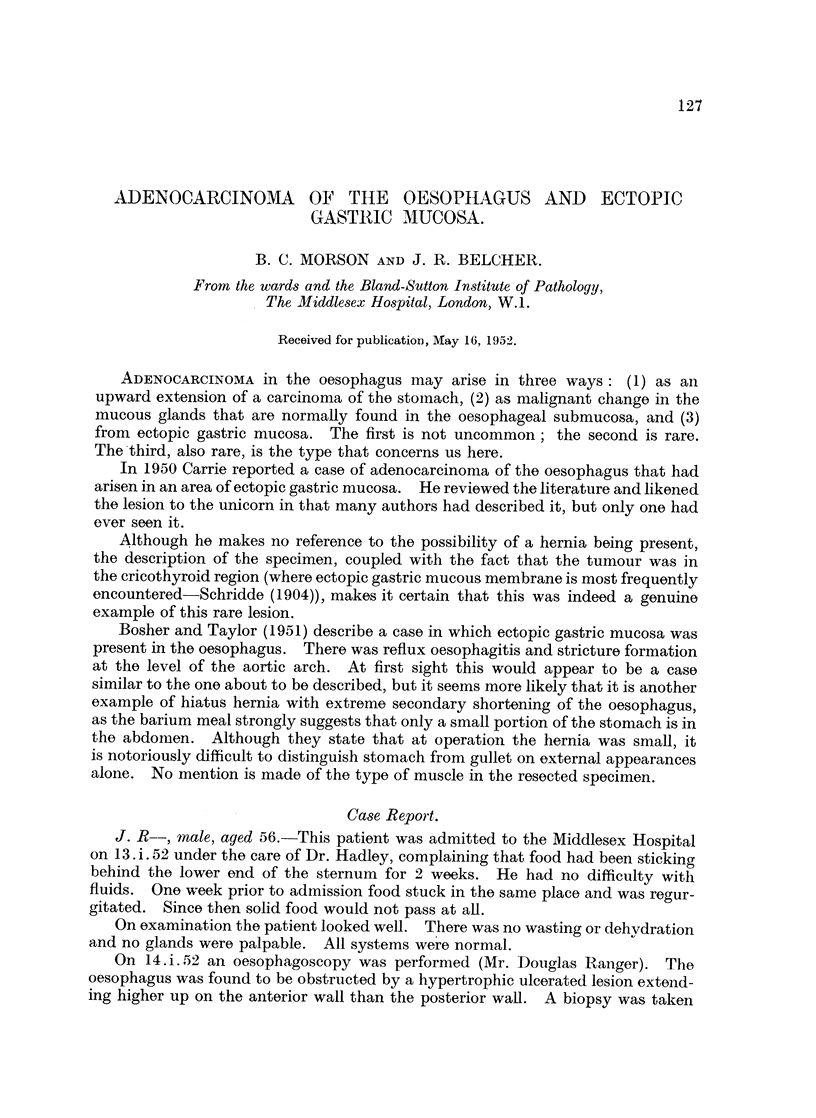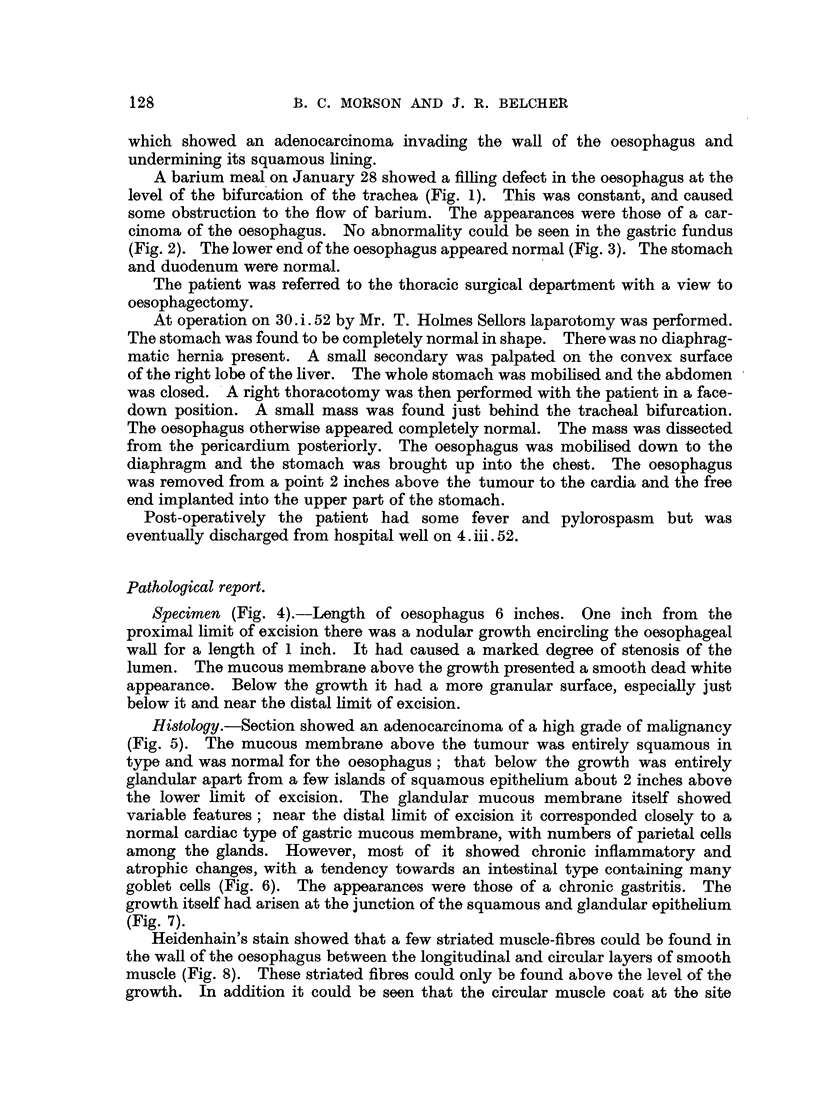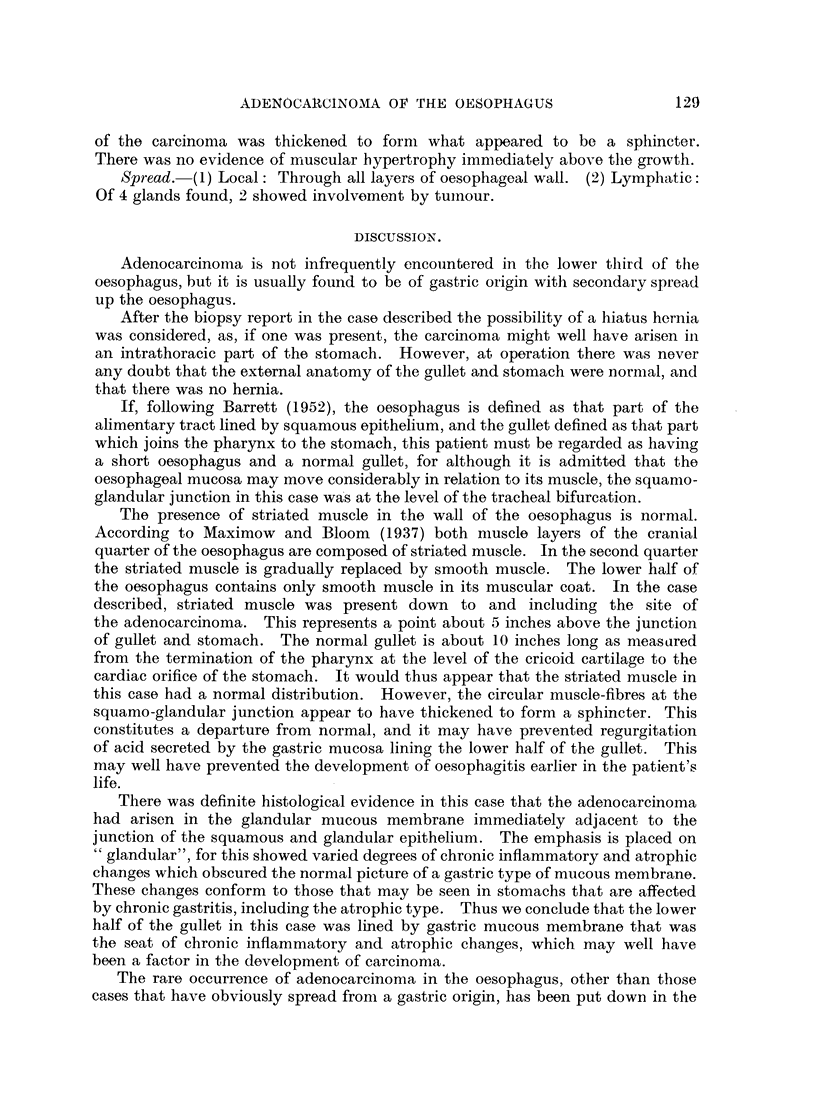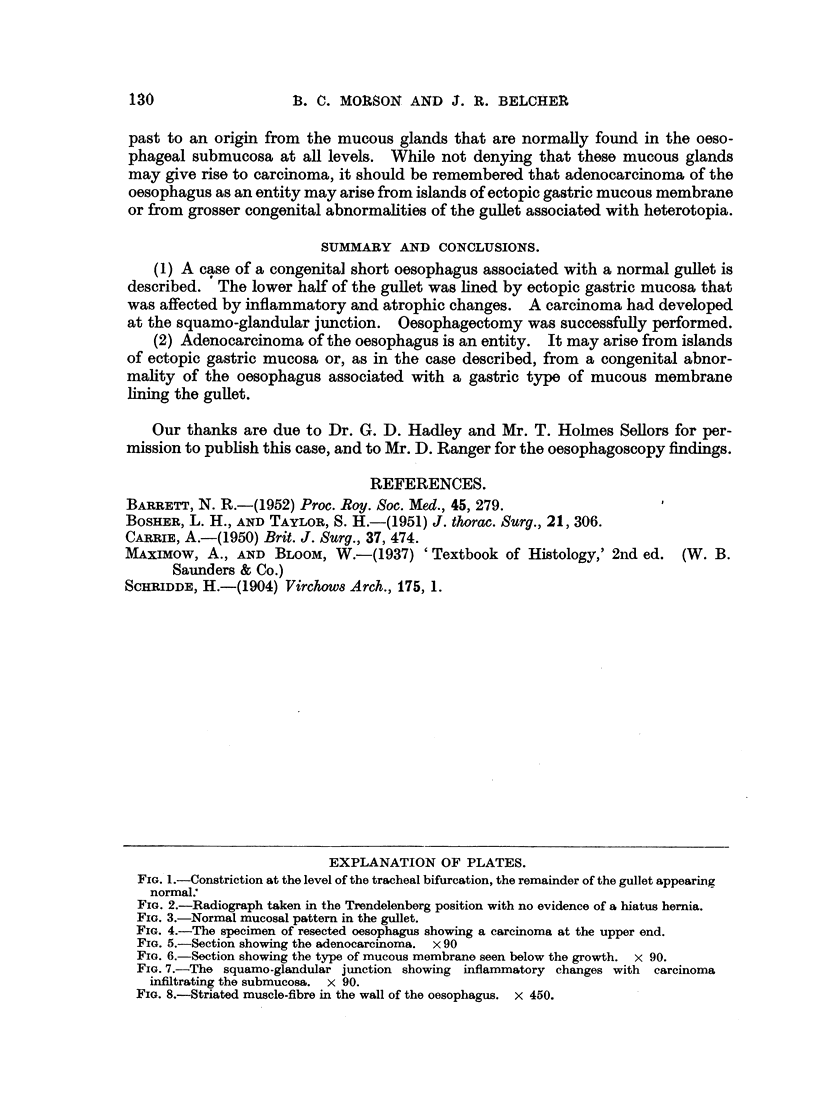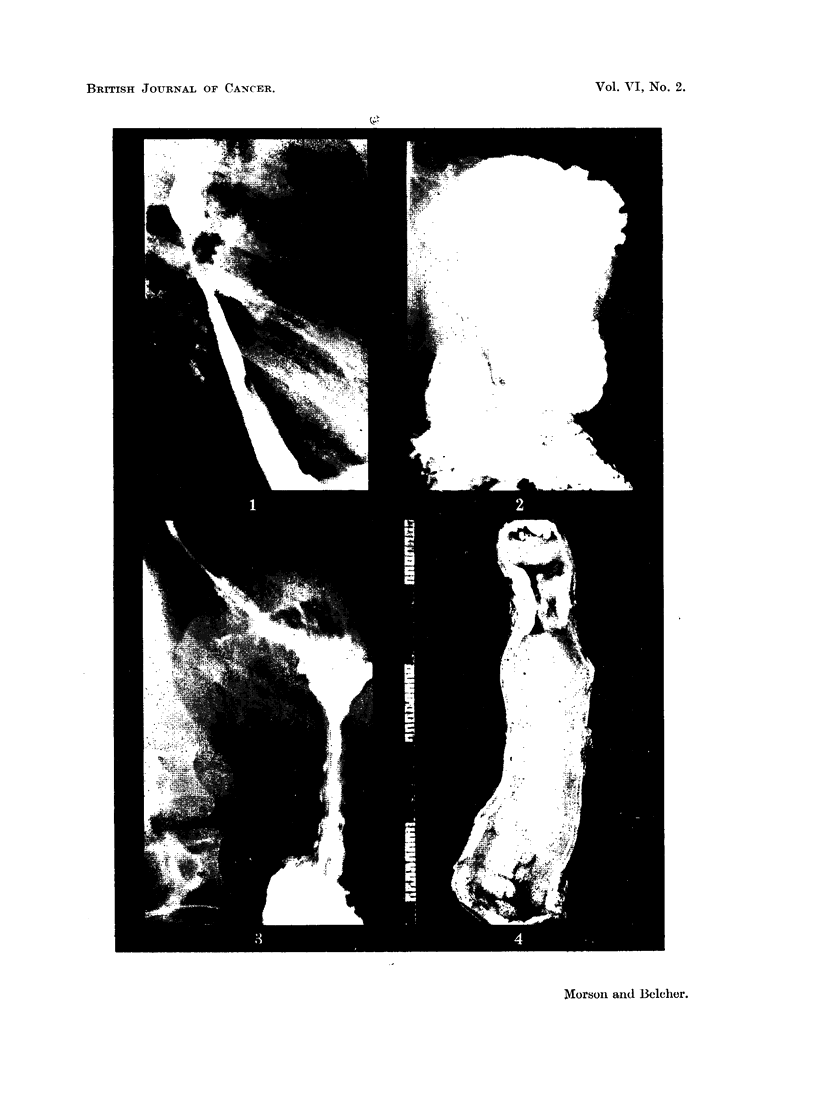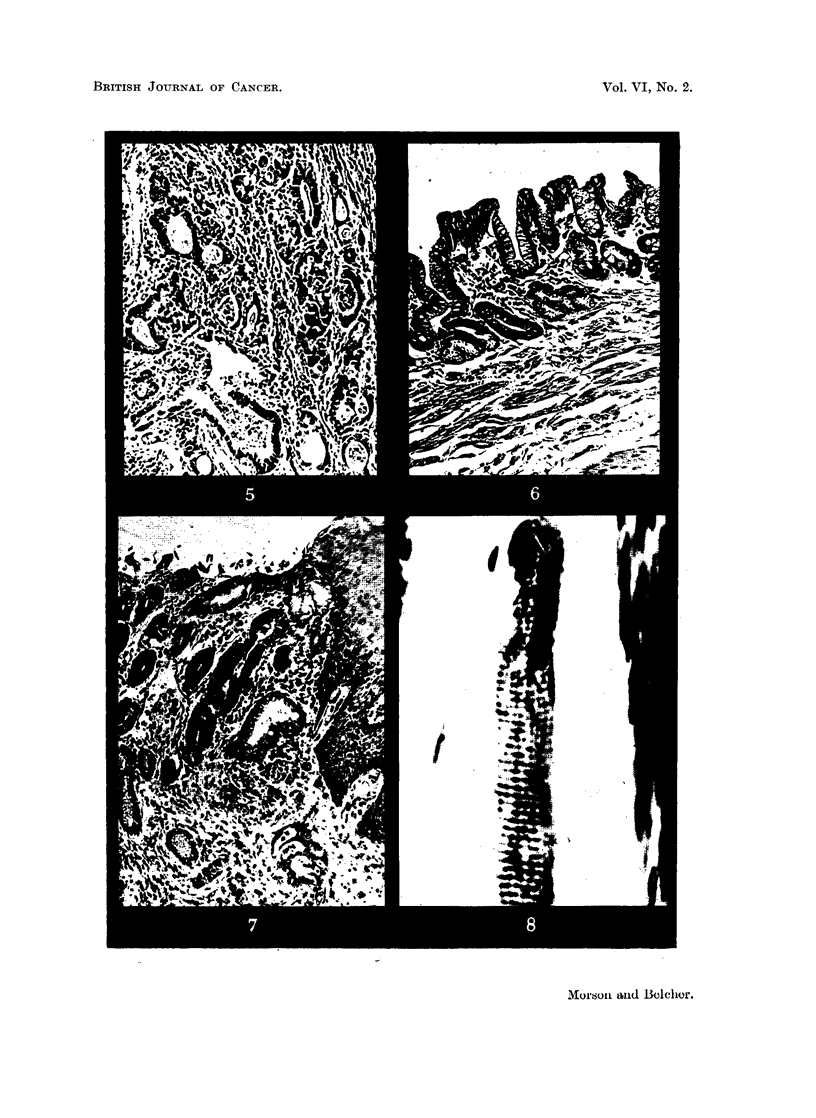# Adenocarcinoma of the Oesophagus and Ectopic Gastric Mucosa

**DOI:** 10.1038/bjc.1952.14

**Published:** 1952-06

**Authors:** B. C. Morson, J. R. Belcher

## Abstract

**Images:**


### ADENOCARCINOMA OF THE OESOPHAGUS AND ECTOPIC GASTRIC MUCOSA.

B. C. MORSON AND J. R. BELCHER.

*From the wards and the Bland-Sutton Institute of Pathology, The Middlesex Hospital, London,* W.1.

Received for publication, May 16, 1952.

ADENOCARCINOMA in the oesophagus may arise in three ways: (1) as an upward extension of a carcinoma of the stomach, (2) as malignant change in the mucous glands that are normally found in the oesophageal submucosa, and (3) from ectopic gastric mucosa. The first is not uncommon; the second is rare. The third, also rare, is the type that concerns us here.

In 1950 Carrie reported a case of adenocarcinoma of the oesophagus that had arisen in an area of ectopic gastric mucosa. He reviewed the literature and likened the lesion to the unicorn in that many authors had described it, but only one had ever seen it.

Although he makes no reference to the possibility of a hernia being present, the description of the specimen, coupled with the fact that the tumour was in the cricothyroid region (where ectopic gastric mucous membrane is most frequently encountered—Schridde (1904)), makes it certain that this was indeed a genuine example of this rare lesion.

Bosher and Taylor (1951) describe a case in which ectopic gastric mucosa was present in the oesophagus. There was reflux oesophagitis and stricture formation at the level of the aortic arch. At first sight this would appear to be a case similar to the one about to be described, but it seems more likely that it is another example of hiatus hernia with extreme secondary shortening of the oesophagus, as the barium meal strongly suggests that only a small portion of the stomach is in the abdomen. Although they state that at operation the hernia was small, it is notoriously difficult to distinguish stomach from gullet on external appearances alone. No mention is made of the type of muscle in the resected specimen.

### *Case Report.*

*J. R—, male, aged* 56.—This patient was admitted to the Middlesex Hospital on 13.i.52 under the care of Dr. Hadley, complaining that food had been sticking behind the lower end of the sternum for 2 weeks. He had no difficulty with fluids. One week prior to admission food stuck in the same place and was regurgitated. Since then solid food would not pass at all.

On examination the patient looked well. There was no wasting or dehydration and no glands were palpable. All systems were normal.

On 14.i.52 an oesophagoscopy was performed (Mr. Douglas Ranger). The oesophagus was found to be obstructed by a hypertrophic ulcerated lesion extending higher up on the anterior wall than the posterior wall. A biopsy was taken which showed an adenocarcinoma invading the wall of the oesophagus and undermining its squamous lining.

A barium meal on January 28 showed a filling defect in the oesophagus at the level of the bifurcation of the trachea (Fig. 1). This was constant, and caused some obstruction to the flow of barium. The appearances were those of a carcinoma of the oesophagus. No abnormality could be seen in the gastric fundus (Fig. 2). The lower end of the oesophagus appeared normal (Fig. 3). The stomach and duodenum were normal.

The patient was referred to the thoracic surgical department with a view to oesophagectomy.

At operation on 30.i.52 by Mr. T. Holmes Sellors laparotomy was performed. The stomach was found to be completely normal in shape. There was no diaphragmatic hernia present. A small secondary was palpated on the convex surface of the right lobe of the liver. The whole stomach was mobilised and the abdomen was closed. A right thoracotomy was then performed with the patient in a face-down position. A small mass was found just behind the tracheal bifurcation. The oesophagus otherwise appeared completely normal. The mass was dissected from the pericardium posteriorly. The oesophagus was mobilised down to the diaphragm and the stomach was brought up into the chest. The oesophagus was removed from a point 2 inches above the tumour to the cardia and the free end implanted into the upper part of the stomach.

Post-operatively the patient had some fever and pylorospasm but was eventually discharged from hospital well on 4.iii.52.

*Pathological report.*

*Specimen* (Fig. 4).—Length of oesophagus 6 inches. One inch from the proximal limit of excision there was a nodular growth encircling the oesophageal wall for a length of 1 inch. It had caused a marked degree of stenosis of the lumen. The mucous membrane above the growth presented a smooth dead white appearance. Below the growth it had a more granular surface, especially just below it and near the distal limit of excision.

*Histology.*—Section showed an adenocarcinoma of a high grade of malignancy (Fig. 5). The mucous membrane above the tumour was entirely squamous in type and was normal for the oesophagus ; that below the growth was entirely glandular apart from a few islands of squamous epithelium about 2 inches above the lower limit of excision. The glandular mucous membrane itself showed variable features ; near the distal limit of excision it corresponded closely to a normal cardiac type of gastric mucous membrane, with numbers of parietal cells among the glands. However, most of it showed chronic inflammatory and atrophic changes, with a tendency towards an intestinal type containing many goblet cells (Fig. 6). The appearances were those of a chronic gastritis. The growth itself had arisen at the junction of the squamous and glandular epithelium (Fig. 7).

Heidenhain's stain showed that a few striated muscle-fibres could be found in the wall of the oesophagus between the longitudinal and circular layers of smooth muscle (Fig. 8). These striated fibres could only be found above the level of the growth. In addition it could be seen that the circular muscle coat at the site of the carcinoma was thickened to form what appeared to be a sphincter. There was no evidence of muscular hypertrophy immediately above the growth.

*Spread.*—(1) Local: Through all layers of oesophageal wall. (2) Lymphatic: Of 4 glands found, 2 showed involvement by tumour.

DISCUSSION.

Adenocarcinoma is not infrequently encountered in the lower third of the oesophagus, but it is usually found to be of gastric origin with secondary spread up the oesophagus.

After the biopsy report in the case described the possibility of a hiatus hernia was considered, as, if one was present, the carcinoma might well have arisen in an intrathoracic part of the stomach. However, at operation there was never any doubt that the external anatomy of the gullet and stomach were normal, and that there was no hernia.

If, following Barrett (1952), the oesophagus is defined as that part of the alimentary tract lined by squamous epithelium, and the gullet defined as that part which joins the pharynx to the stomach, this patient must be regarded as having a short oesophagus and a normal gullet, for although it is admitted that the oesophageal mucosa may move considerably in relation to its muscle, the squamo-glandular junction in this case was at the level of the tracheal bifurcation.

The presence of striated muscle in the wall of the oesophagus is normal. According to Maximow and Bloom (1937) both muscle layers of the cranial quarter of the oesophagus are composed of striated muscle. In the second quarter the striated muscle is gradually replaced by smooth muscle. The lower half of the oesophagus contains only smooth muscle in its muscular coat. In the case described, striated muscle was present down to and including the site of the adenocarcinoma. This represents a point about 5 inches above the junction of gullet and stomach. The normal gullet is about 10 inches long as measured from the termination of the pharynx at the level of the cricoid cartilage to the cardiac orifice of the stomach. It would thus appear that the striated muscle in this case had a normal distribution. However, the circular muscle-fibres at the squamo-glandular junction appear to have thickened to form a sphincter. This constitutes a departure from normal, and it may have prevented regurgitation of acid secreted by the gastric mucosa lining the lower half of the gullet. This may well have prevented the development of oesophagitis earlier in the patient's life.

There was definite histological evidence in this case that the adenocarcinoma had arisen in the glandular mucous membrane immediately adjacent to the junction of the squamous and glandular epithelium. The emphasis is placed on "glandular", for this showed varied degrees of chronic inflammatory and atrophic changes which obscured the normal picture of a gastric type of mucous membrane. These changes conform to those that may be seen in stomachs that are affected by chronic gastritis, including the atrophic type. Thus we conclude that the lower half of the gullet in this case was lined by gastric mucous membrane that was the seat of chronic inflammatory and atrophic changes, which may well have been a factor in the development of carcinoma.

The rare occurrence of adenocarcinoma in the oesophagus, other than those cases that have obviously spread from a gastric origin, has been put down in the past to an origin from the mucous glands that are normally found in the oesophageal submucosa at all levels. While not denying that these mucous glands may give rise to carcinoma, it should be remembered that adenocarcinoma of the oesophagus as an entity may arise from islands of ectopic gastric mucous membrane or from grosser congenital abnormalities of the gullet associated with heterotopia.

### SUMMARY AND CONCLUSIONS.

(1) A case of a congenital short oesophagus associated with a normal gullet is described. The lower half of the gullet was lined by ectopic gastric mucosa that was affected by inflammatory and atrophic changes. A carcinoma had developed at the squamo-glandular junction. Oesophagectomy was successfully performed.

(2) Adenocarcinoma of the oesophagus is an entity. It may arise from islands of ectopic gastric mucosa or, as in the case described, from a congenital abnormality of the oesophagus associated with a gastric type of mucous membrane lining the gullet.

Our thanks are due to Dr. G. D. Hadley and Mr. T. Holmes Sellors for permission to publish this case, and to Mr. D. Ranger for the oesophagoscopy findings.

### REFERENCES.

Barrett, N. R.—(1952) *Proc. Roy. Soc. Med.*, **45**, 279.
Bosher, L. H., and Taylor, S. H.—(1951) *J. thorac. Surg.*, **21**, 306.
Carrie, A.—(1950) *Brit. J. Surg.*, **37**, 474.
Maximow, A., and Bloom, W.—(1937) 'Textbook of Histology,' 2nd ed. (W. B. Saunders & Co.)
Schridde, H.—(1904) *Virchows Arch.*, **175**, 1.

### EXPLANATION OF PLATES.

Fig. 1.—Constriction at the level of the tracheal bifurcation, the remainder of the gullet appearing normal.
Fig. 2.—Radiograph taken in the Trendelenberg position with no evidence of a hiatus hernia.
Fig. 3.—Normal mucosal pattern in the gullet.
Fig. 4.—The specimen of resected oesophagus showing a carcinoma at the upper end.
Fig. 5.—Section showing the adenocarcinoma. × 90
Fig. 6.—Section showing the type of mucous membrane seen below the growth. × 90.
Fig. 7.—The squamo-glandular junction showing inflammatory changes with carcinoma infiltrating the submucosa. × 90.
Fig. 8.—Striated muscle-fibre in the wall of the oesophagus. × 450.